# Identification of a compound heterozygote in *LYST* gene: a case report on Chediak-Higashi syndrome

**DOI:** 10.1186/s12881-019-0922-8

**Published:** 2020-01-06

**Authors:** Yinsen Song, Zhengping Dong, Shuying Luo, Junmei Yang, Yuebing Lu, Bo Gao, Tianli Fan

**Affiliations:** 1 Central Laboratory, Zhengzhou People’s Hospital Affiliated to Southern Medical University, Children’s Hospital Affiliated to Zhengzhou University, Zhengzhou, China; 2 Binzhou medical University, Yantai, China; 3 Department of Oncology, Children’s Hospital Affiliated to Zhengzhou University, Zhengzhou, China; 4 Department of Laboratory Medicine, Taihe Hopsital, Hubei University of Medicine, Shiyan, China; 5 School of Basic Medical Sciences, Zhengzhou University, Zhengzhou, 450001 Henan China

**Keywords:** Chediak-Higashi syndrome, *LYST* gene, Compound heterozygote, Amplicon sequencing, Two-generation pedigree

## Abstract

**Background:**

Chediak-Higashi Syndrome (CHS) is a rare autosomal recessive disease caused by loss of function of the lysosomal trafficking regulator protein. The causative gene *LYST/CHS1* was cloned and identified in 1996, which showed significant homology to other species such as bovine and mouse. To date, 74 pathogenic or likely pathogenic mutations had been reported.

**Case presentation:**

Here we describe a compound heterozygote in *LYST* gene, which was identified in a 4-year-old female patient. The patient showed skin hypopigmentation, sensitivity to light, mild splenomegaly and reduction of platelets in clinical examination. Giant intracytoplasmic inclusions were observed in the bone marrow examination, suggesting the diagnosis of CHS. Amplicon sequencing was performed to detect pathogenic mutation in LYST gene. The result was confirmed by two-generation pedigree analysis base on sanger sequencing.

**Conclusion:**

A compound heterozygote in *LYST* gene, consisting of a missense mutation c.5719A > G and an intron mutation c.4863-4G > A, was identified from the patient by using amplicon sequencing. The missense mutation is reported for the first time. Two-generation pedigree analysis showed these two mutations were inherited from the patient’s parents, respectively. Our result demonstrated that amplicon sequencing has great potential for accelerating and improving the diagnosis of rare genetic diseases.

## Background

Chediak-Higashi Syndrome (CHS, MIM: 214500) rare but lethal autosomal recessive disorder characterized by multiple clinical features, including hypopigmentation of skin and hair, reduction of platelets and leukocytes, abnormal organelles in circulating granulated cells, and neurological dysfunction [1, 2]. Recurrent infections could also be observed due to severe immunodeficiency. The primary immunologic symptom lymphoproliferative histiocytosis, which called the accelerated phase determines the prognosis of CHS [3]. Patients with early-onset accelerated phase (before the age of 6) often have severe phenotype and died in the first decade, however, about 10–15% of patients with the late-onset forms follow a less severe clinical course of CHS and survive without having treatment of bone marrow transplantation [4, 5].

CHS was first described in 1950s [6], but the causative gene had not been revealed until 1996 [7]. The *LYST* gene (HGNC:1968), consisting of 53 exons with a mRNA transcript of 13,503 bp, was identified to be responsible for this disease [8, 9]. Previous studies suggested that the product of the *LYST* gene is required for sorting endosomal resident proteins into late multivesicular endosomes [10]. To date, 74 pathogenic or likely pathogenic mutations in *LYST* gene had been reported (Additional file 2: Table S1), correlations between genotypes and phenotypes had been investigated.

Here we describe a compound heterozygote in *LYST* gene, consisting of a missense mutation NM_000081.2:c.5719A > G (p.Ile1907Val) and a intron mutation NC_000001.10:g.235945391C > T (c.4863-4G > A), which was identified in a 4-year-old female patient with CHS. Mutations in *LYST* gene were detected by using amplicon sequencing within 24 h. We also performed sanger sequencing to confirm the result and identify the source of mutations in the patient’s family. A typical autosomal recessive inheritance pattern was demonstrated by our data. Our finding expands the spectrum of pathogenic mutations in *LYST* gene and indicates an efficient and accurate approach for diagnosis of genetic diseases.

## Case presentation

The 4-year-old female patient was admitted to Children’s Hospital Affiliated to Zhengzhou University with recurrent upper respiratory infections last for 2 months. Antibiotic treatment had been taken but without any improvement. Patient and her parents are all Han Chinese from Henan province of China, no family history was noticed in records. The study was approved by the ethics committee of Children’s Hospital Affiliated to Zhengzhou University. A written informed consent for publishing data and images was obtained from the parents before performing genetic test.

**Fig. 1:**
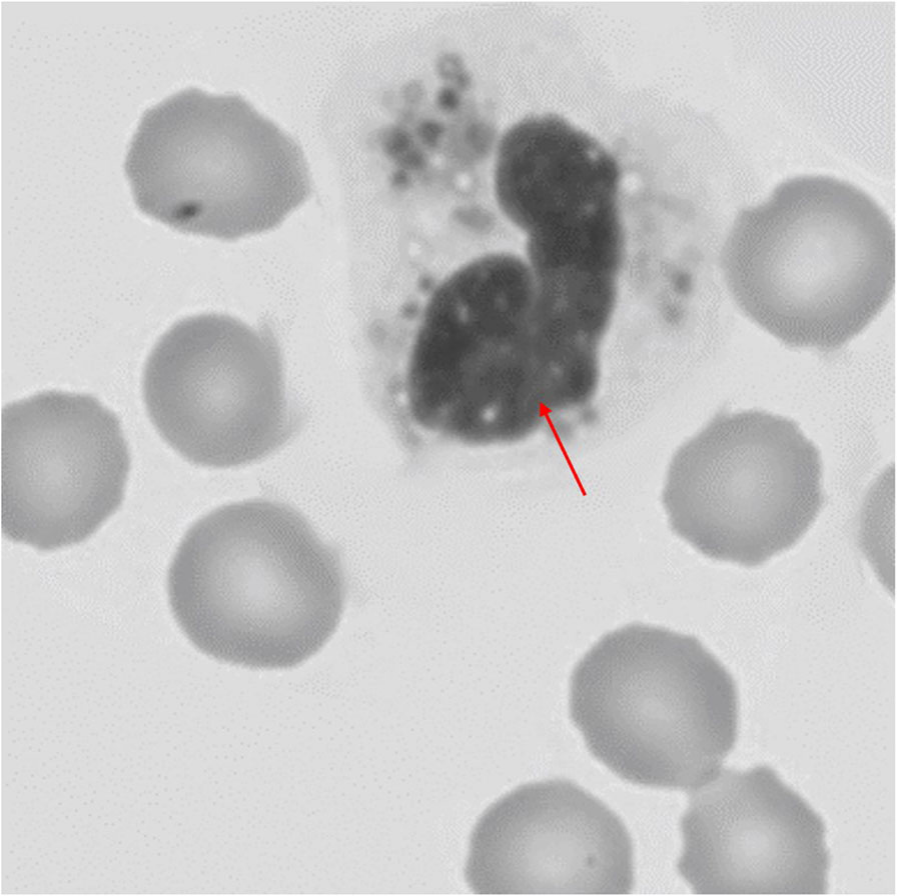
Bone marrow examination for patient with CHS. Giant intracytoplasmic inclusion in leukocyte is indicated by red arrow

The patient had low fever and cough, temperature was measured to 38.5 °C on regular examination. Hypopigmentation of skin on neck and knee was observed on physical examination. Slight hepatomegaly was detected by supersonic inspection. Blood analysis showed reduction of platelets (PLT, 88*10^9^/L) and white blood cell (WBC, 2.7*10^9^/L), the red blood cell was normal (RBC, 4.9*10^12^/L). Leukocytes with giant intracytoplasmic inclusions were observed with peripheral smear, which evidenced by bone marrow aspirate (Fig. 1). The neurological examination was normal. Clinical manifestations suggested the diagnosis of CHS. Genetic test for the patient and her parents was recommended.

## Identification of causative mutations in *LYST* gene

A customized panel covering all 53 exons and 10 bp of padding region of *LYST* gene was designed by using the online tool (https://www.ampliseq.com), details was described in Additional file 2: Table S1. Genomic DNA was purified from peripheral blood mononuclear cells (PBMC) with widely used commercial kit (Tiangen Biotech, Beijing, China) according to manufacturer’s protocol. DNA library was prepared by multiple PCR in one tube and sequenced with the Ion S5XL genetic analyzer (ThermoFisher Scientific, Waltham, MA, USA). Mutations were detected by using VariantCaller V1.0, along with human genome reference hg19 (GRCh37). Clinical significance of mutations was predicted by using SIFT (Version 5.11) [11] and Human Splicing Finder (Version 3.1) [12].

**Fig. 2:**
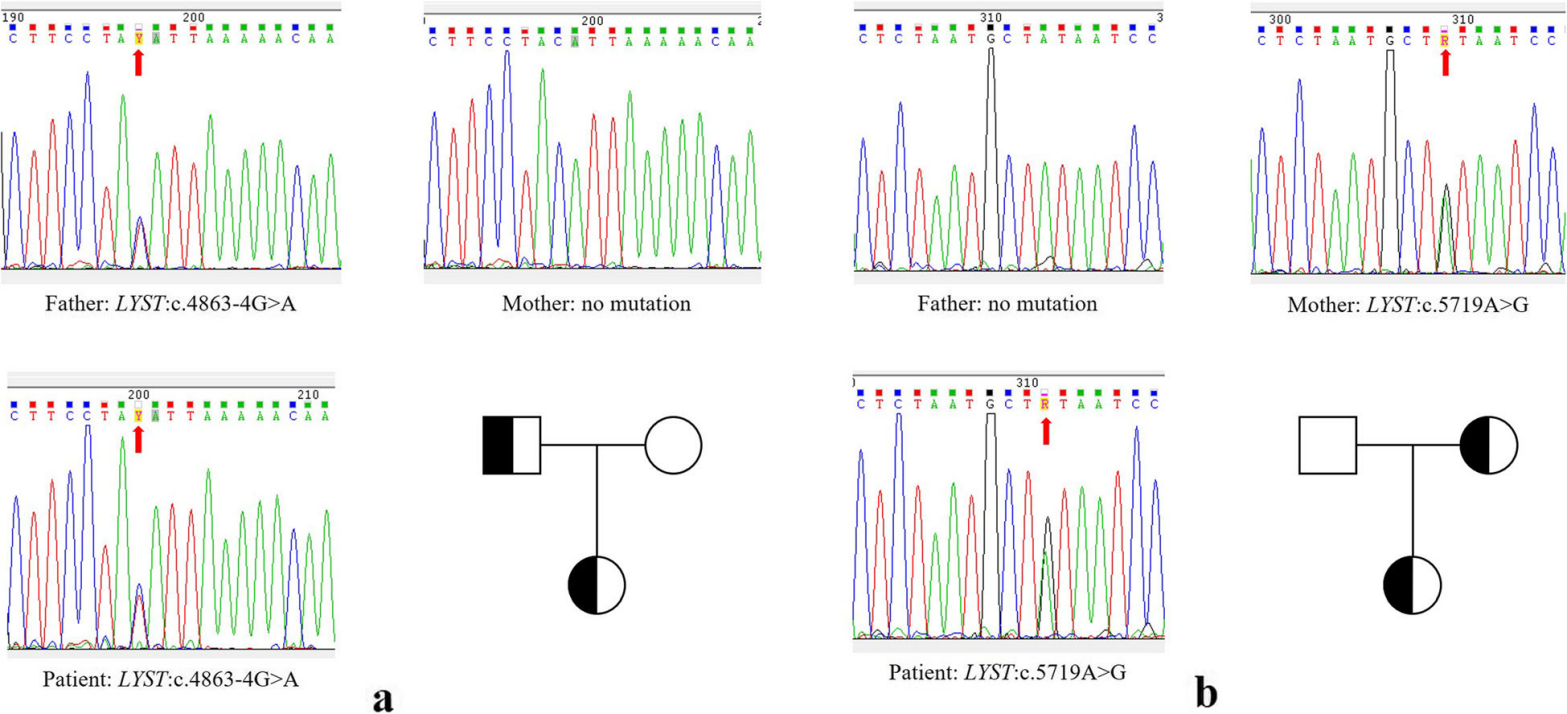
Identified variations in *LYST* gene. a. Reported intron mutation (c.4863-4G > A) in exon15, inherited from father. b. Novel missense mutation (c.5719A > G) in exon19, inherited from mother. Mutations are marked with red arrow. Zygosity is showed by letters of degenerate bases (Y for C/T, R for A/G)

As result, two heterozygous mutations were detected, including one missense mutation NM_000081.2:c.5719A > G (p.Ile1907Val) and a intron mutation NC_000001.10:g.235945391C > T (c.4863-4G > A) (Table 1). The intron mutation c.4863-4G > A is a known SNP (rs201382097) which distributes mainly in Asian (Additional file 3: Table S2 and Additional file 4: Table S3), our data indicates likely pathogenic by affect splicing (Additional file 1). The missense mutation c.5719A > G locates in exon19 of *LYST* gene, resulting an amino acid sequence change of I1907V, is reported for the first time and predicted to be damaging for protein function by SIFT. The result was confirmed by sanger sequencing. Two-generation pedigree analysis was performed, targeting these two mutations. A typical autosomal recessive inheritance pattern was demonstrated, the missense mutation c.5719A > G was inherited from patient’s mother, the other was from father (Fig. 2).

**Table 1:** Identified variations from the patient with CHS

Variation	Location	Peptide change	Type	Zygosity	ID in dbSNP	Allele Frequency in gnomAD
NM_000081.2: c.5719A > G	Chr1:235937207	p.Ile1907Val	missense	Heterozygous	not applicable	not applicable
NC_000001.10: g.235945391C > T	Chr1: 235945391	p.=	intron	Heterozygous	rs201382097	0.0006894
NM_003664.3: c.1754 T > A	Chr5:77425028	p.Val585Glu	missense	Heterozygous	rs6453373	0.8668
NM_001083116.1: c.900C > T	Chr10:72358577	p.=	synonymous	Heterozygous	rs885822	0.6403
NM_199242.2: c.3198A > G	Chr17:73824121	p.=	synonymous	Heterozygous	rs7210574	0.3966
NM_199242.2: c.2599A > G	Chr17:73827205	p.Lys867Glu	missense	Heterozygous	rs1135688	0.3626
NM_199242.2: c.1977C > T	Chr17:73831016	p.=	synonymous	Heterozygous	rs2290770	0.02977
NM_199242.2: c.888G > C	Chr17:73836162	p.=	synonymous	Heterozygous	rs3744026	0.1496
NM_199242.2: c.279C > T	Chr17:73839137	p.=	synonymous	Heterozygous	rs3744007	0.04832
NM_006949.2: c.38-7C > T	Chr19:7703605	p.=	intron	Heterozygous	rs8104339	0.4047
NM_006949.2: c.1443 T > C	Chr19:7711221	p.=	synonymous	Heterozygous	rs10001	0.4204
NM_006949.2: c.1576A > G	Chr19:7712277	p.Ile526Val	missense	Heterozygous	rs6791	0.6401
NM_001204401.1: c.1268A > C	ChrX:123034511	p.Gln423Pro	missense	Heterozygous	rs5956583	0.3378

**Fig. 3:**
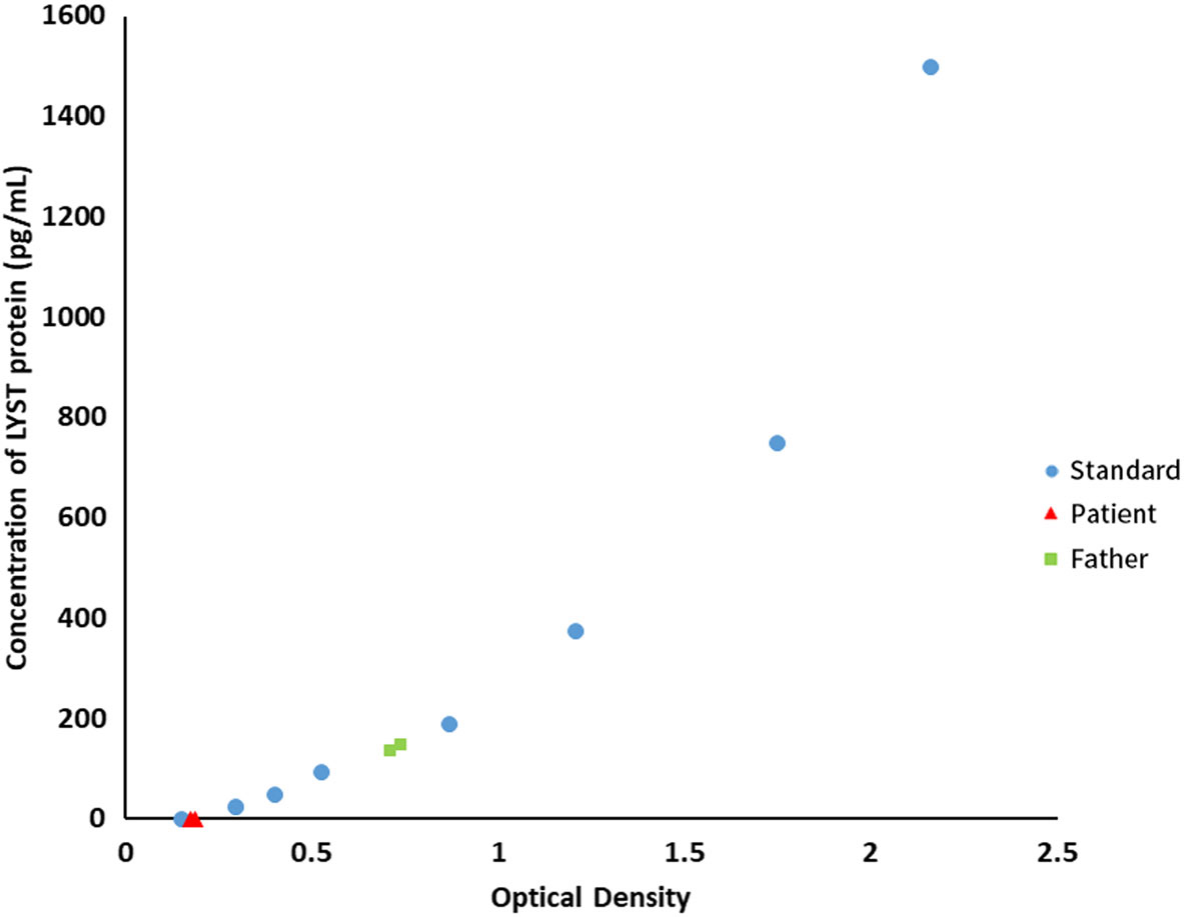
ELISA test for human LYST protein. Dot: Standards provided in the ELISA kit. Triangle: Plasma sample from the patient. Square: Plasma sample from the patient’s father

To further confirm the influence caused by these two mutations, an ELISA test for LYST protein was performed using commercial ELISA kit purchased from Cusabio Bioengineering co. LTD (Wuhan, China). 400 μL of plasma from the patient and her father was used to extract protein, then tested according to manufacturer’s protocol. The result showed that normal LYST protein is undetectable in plasma of the patient, proving the loss of function of LYST protein (Fig. 3).

## Discussion and conclusions

Here we described a compound heterozygote in *LYST* gene identified from a 4-year-old female patient in China, who was diagnosed with CHS based on clinical manifestations including hypopigmentation of skin, hepatomegaly, reduction of PLT and WBC, and leukocytes with giant intracytoplasmic inclusions. Genetic test showed two heterozygous mutations were inherited from her father and mother, respectively. The girl was treated with enhanced anti-infective therapy and liver protection, the clinical manifestation was stabilized. Further treatment including HSCT will be evaluated based on conditions of prognosis.

To date, 74 pathogenic or likely pathogenic mutations in *LYST* gene had been reported in ClinVar database (https://www.ncbi.nlm.nih.gov/clinvar/) (Additional file 5: Table S4), the genotype-phenotype correlations had been investigated in the past two decades [9]. In general, loss-of-function variants such as nonsense or frame-shift mutations are associated with severe, childhood-onset form and missense variants with milder adolescent- or adult-onset forms of the disorder [4, 13]. In this case, a compound heterozygote consisting of a missense and an intron mutation was identified. The intron mutation c.4863-4G > A was predicted to be probably affecting splicing, might lead to loss-of-function and cause severe conditions. In this case, phenotype of the patient was mild and stabilized for now, but long-term nursing is necessary to monitor the onset of accelerated phase. However, a conflict record had been submitted to ClinVar database by single submitter. According to the dbSNP and 1000 genomes database, this mutation mainly distributes in Asian people, especially in southwest of China and Southeast Asia. Therefore, further studies are required to confirm the clinical significance of the mutation. On the other hand, a significant disadvantage of exon sequencing should not be neglected, that is the cost-saving method is unable to detect mutations in deep intron. In this report, we couldn’t eliminate the possibility that there is other causative mutation locates in deep intron of the *LYST* gene. A whole-genome sequencing should be performed in further study with the permission of the patient and her family.

Molecular diagnosis for CHS could benefit not only treatment and prognosis, but also differential diagnosis. There are several diseases resemble CHS, including Hermansky Pudlak syndrome 2 (HPS2, MIM: 608233), Griscelli syndrome (GS, OMIM Phenotypic Series: PS214450) and Familial hemophagocytic lymphohistiocytosis (FHL, OMIM Phenotypic Series: PS267700). Amplicon sequencing based on ultrahigh-efficient multiplex PCR provides an approach for detecting mutations from targeted regions on genome. The whole process of DNA preparing, sequencing and data analyzing could be finished in 24 h, which could improve the efficiency in the clinical laboratories.

In summary, a compound heterozygote in *LYST* gene was identified from a 4-year-old female patient with CHS, a novel missense mutation was included. The result was confirmed by sanger sequencing. The source of mutations was investigated by two-generation pedigree analysis. Our data expands the spectrum of pathogenic mutations in *LYST* gene, demonstrates the value of amplicon sequencing as an efficient and accurate approach for diagnosis of genetic diseases.

## Supplementary information

**Additional file 1: **Functional prediction of mutations performed by HSF 3.1, including the novel missense *LYST:*c.5719A > G, the known SNP rs201382097 (*LYST*:c.4863-4G > A), and two mutations in adjacent sites of rs201382097, which referred to *LYST*:c.4863-3 T > A and *LYST*:c.4863-5 T > A.

**Additional file 2: Table S1.** Information of custom-designed amplicon sequencing panel for CHS and related disorders.

**Additional file 3: Table S2.** Global distribution of the mutation *LYST*:c.4863-4G>A (rs201382097) in gnomAD database.

**Additional file 4: Table S3.** Global distribution of the mutation *LYST*:c.4863-4G>A (rs201382097) in 1000 genome database.

**Additional file 5: Table S4.** Reported pathogenic and likely pathogenic mutations in the LYST gene from the ClinVar database.

## Data Availability

The sequence data had been deposited into NCBI Short Read Archive under accession number SRR10382506.

## Abbreviations

CHS: Chediak-Higashi Syndrome
HSCT: Hematopoietic stem cell transplantation
PBMC: Peripheral blood mononuclear cells
PLT: Platelets
RBC: Red blood cell
WBC: White blood cell
