# Supplementary material for: Identification of a compound heterozygote in LYST gene: a case report on Chediak-Higashi syndrome
Source: BMC Med Genet. 2020 Jan 6;21:4. doi: 10.1186/s12881-019-0922-8 (PMC6943916; doi:10.1186/s12881-019-0922-8)

**Additional file**

Functional influence of mutations in LYST gene was predicted by human splicing finder (HSF, version 3.1. <http://www.umd.be/HSF3>). The novel missense mutation c.5719A>G was predicted to be potential alteration of splicing. The known SNP rs201382097 (*LYST*:c.4863-4G>A) has probably no impact on splicing. However, mutations in adjacent sites of c.4863-4G could be most probably affecting splicing, indicating a potential splice site around the position.


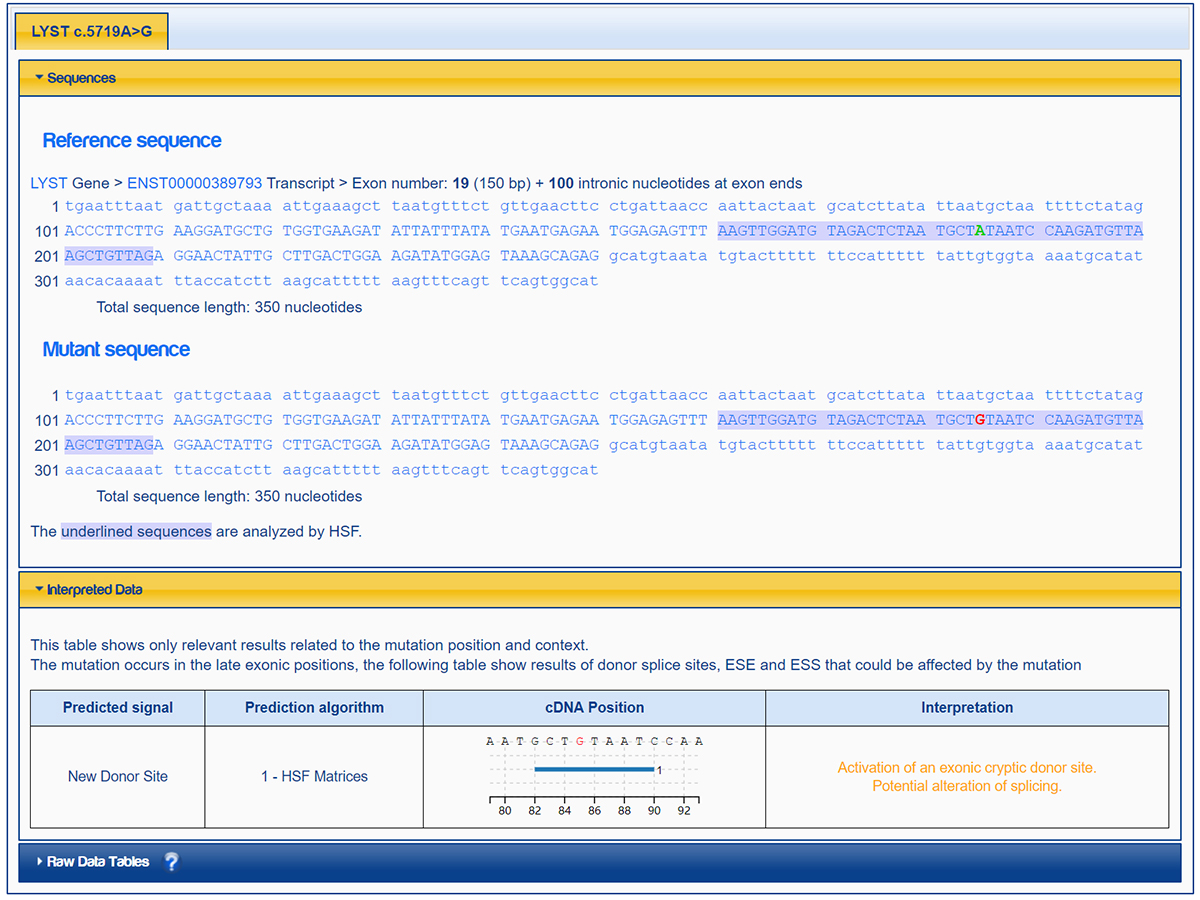

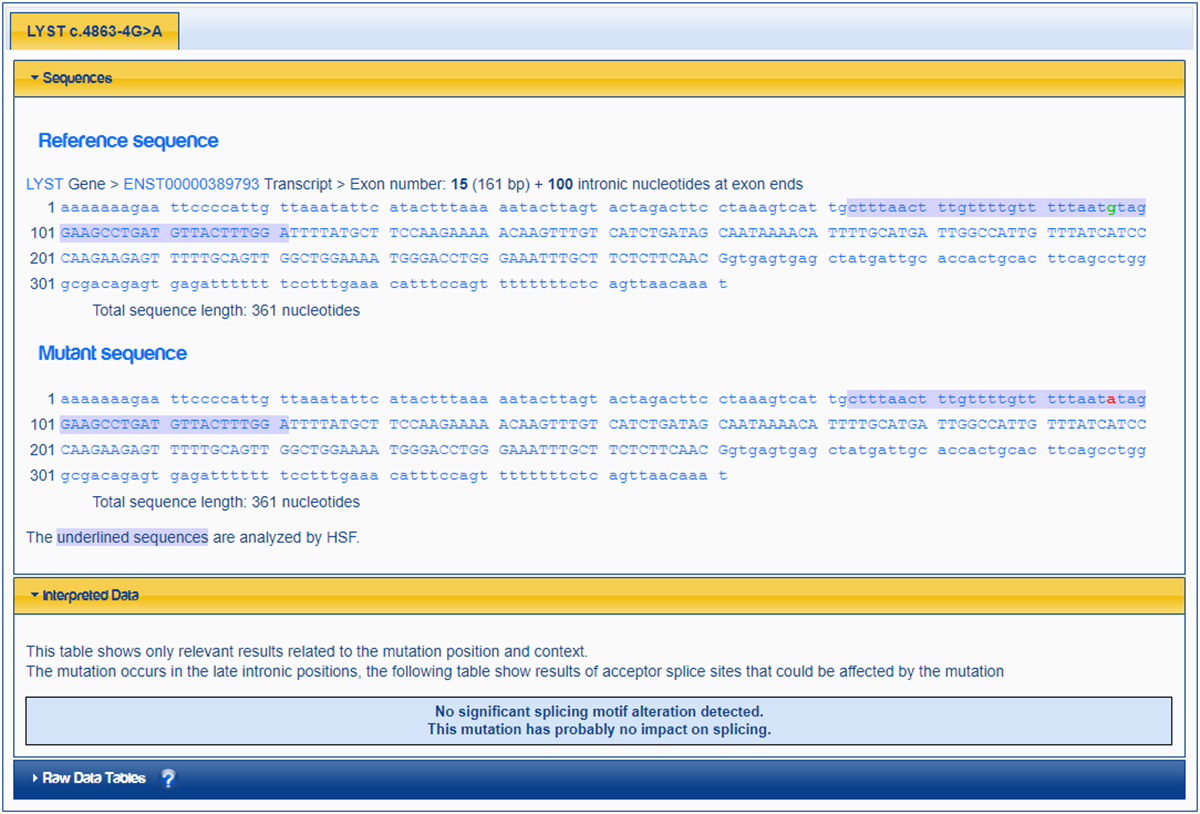

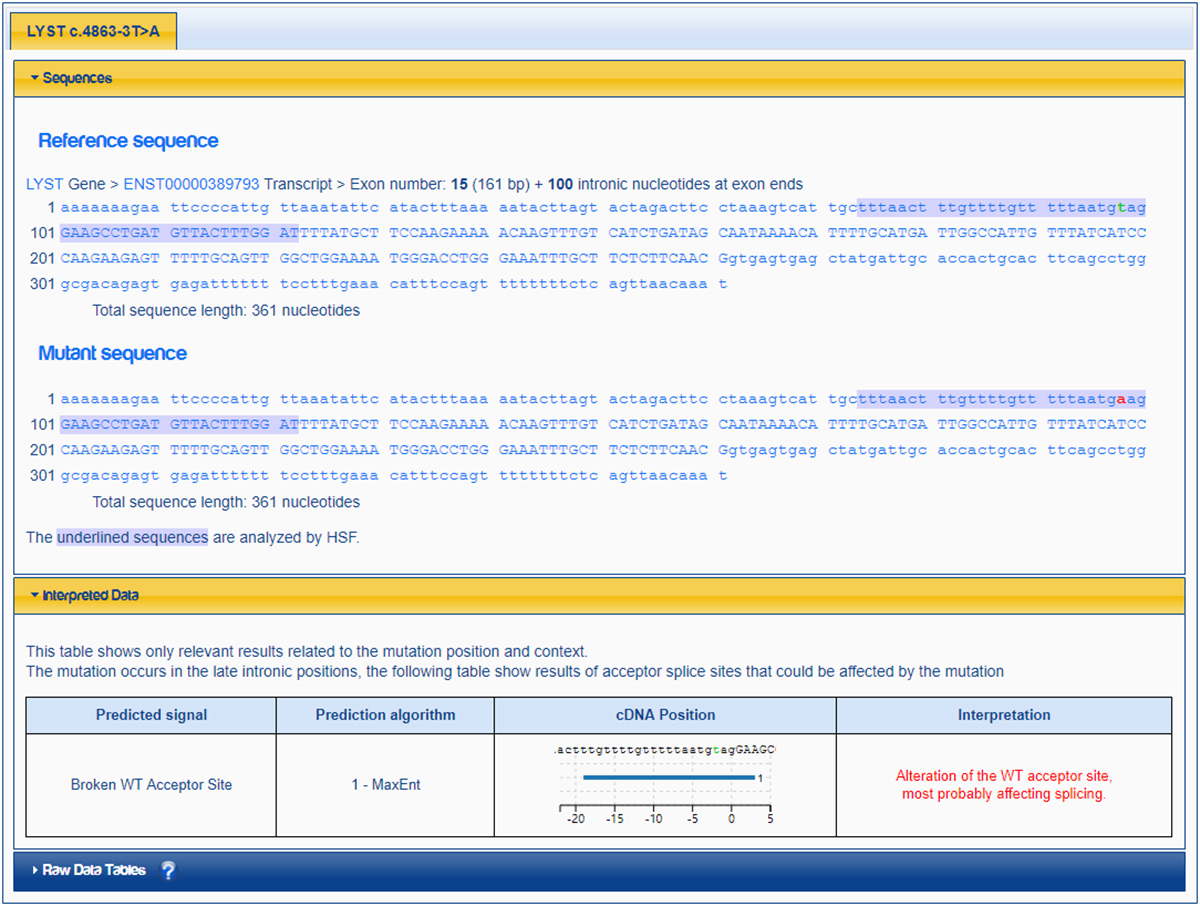

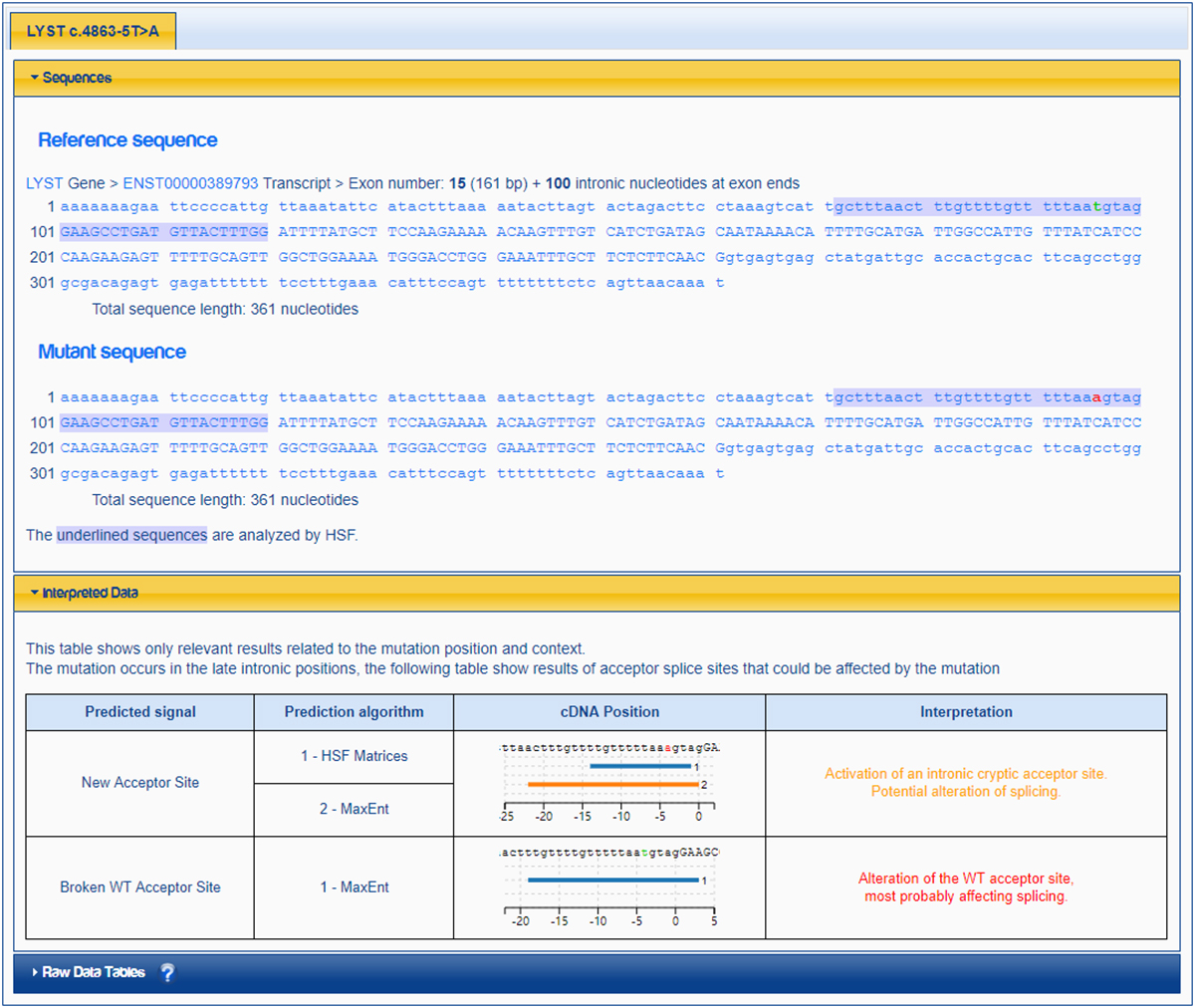

Supplement: Supplementary file 1 — Additional file 1: Functional prediction of mutations performed by HSF 3.1, including the novel missense LYST:c.5719A > G, the known SNP rs201382097 (LYST:c.4863-4G > A), and two mutations in adjacent sites of rs201382097, which referred to LYST:c.4863-3 T > A and LYST:c.4863-5 T > A. [file 12881_2019_922_MOESM1_ESM.docx]
