# Supplementary material for: Identification of a compound heterozygote in LYST gene: a case report on Chediak-Higashi syndrome
Source: BMC Med Genet. 2020 Jan 6;21:4. doi: 10.1186/s12881-019-0922-8 (PMC6943916; doi:10.1186/s12881-019-0922-8)
Supplement: Supplementary file 2 — Additional file 2: Table S1. Information of custom-designed amplicon sequencing panel for CHS and related disorders. [file 12881_2019_922_MOESM2_ESM.docx]

Table S1. Custom-designed amplicon sequencing panel for CHS and related disorders.

| **Gene Symbol** | **HGNC ID** | **Chromosomal Location** | **Whole Exon Length (bp)** | **Amplicon Number** | **Overall Coverage (%)** |
| --- | --- | --- | --- | --- | --- |
| LYST | HGNC:1968 | 1q42.3 | 17,833 | 101 | 99.9 |
| AP3B1 | HGNC:566 | 5q14.1 | 4,589 | 35 | 100 |
| ITK | HGNC:6171 | 5q33.3 | 4,706 | 26 | 100 |
| STX11 | HGNC:11429 | 6q24.2 | 5,544 | 20 | 92.6 |
| PRF1 | HGNC:9360 | 10q22.1 | 2,594 | 13 | 100 |
| RAB27A | HGNC:9766 | 15q21.3 | 3,879 | 18 | 99.5 |
| UNC13D | HGNC:23147 | 17q25.3 | 5,031 | 40 | 94.4 |
| STXBP2 | HGNC:11445 | 19p13.2 | 2,384 | 22 | 92.0 |
| SH2D1A | HGNC:10820 | Xq25 | 2,597 | 12 | 100 |
| XIAP | HGNC:592 | Xq25 | 8,705 | 35 | 83.2 |
| **Total** | **NA** | **NA** | **55,479** | **322** | **95.7** |
