# Supplementary material for: Identification of a compound heterozygote in LYST gene: a case report on Chediak-Higashi syndrome
Source: BMC Med Genet. 2020 Jan 6;21:4. doi: 10.1186/s12881-019-0922-8 (PMC6943916; doi:10.1186/s12881-019-0922-8)
Supplement: Supplementary file 3 — Additional file 3: Table S2. Global distribution of the mutation LYST:c.4863-4G>A (rs201382097) in gnomAD database. [file 12881_2019_922_MOESM3_ESM.docx]

Table S2. Global distribution of NC_000001.10:g.235945391C>T (rs201382097) in gnomAD.

| Populations | Ancestral Allele: C | Minor Allele: T | MAF |
| --- | --- | --- | --- |
| global | 271073 | 187 | 0.0006894 |
| East Asian | 18141 | 183 | 0.009987 |
| South Asian | 30413 | 3 | 9.9×10^-5^ |
| European (Non-Finnish) | 123109 | 1 | 8×10^-6^ |
| African | 23710 | 0 | 0 |
| Ashkenazi Jewish | 10052 | 0 | 0 |
| European (Finnish) | 25250 | 0 | 0 |
| Latino | 34038 | 0 | 0 |
| Other | 6360 | 0 | 0 |
