# Supplementary material for: Identification of a compound heterozygote in LYST gene: a case report on Chediak-Higashi syndrome
Source: BMC Med Genet. 2020 Jan 6;21:4. doi: 10.1186/s12881-019-0922-8 (PMC6943916; doi:10.1186/s12881-019-0922-8)
Supplement: Supplementary file 4 — Additional file 4: Table S3. Global distribution of the mutation LYST:c.4863-4G>A (rs201382097) in 1000 genome database. [file 12881_2019_922_MOESM4_ESM.docx]

Table S3. Global distribution of NC_000001.10:g.235945391C>T (rs201382097) in 1000 genome database.

| *Populations | Ancestral Allele: C | Minor Allele: T | MAF |
| --- | --- | --- | --- |
| global | 4991 | 17 | 0.0034 |
| ACB | 192 | 0 | 0.0000 |
| ASW | 122 | 0 | 0.0000 |
| BEB | 172 | 0 | 0.0000 |
| CDX | 180 | 6 | 0.0323 |
| CEU | 198 | 0 | 0.0000 |
| CHB | 204 | 2 | 0.0097 |
| CHS | 208 | 2 | 0.0095 |
| CLM | 188 | 0 | 0.0000 |
| ESN | 198 | 0 | 0.0000 |
| FIN | 198 | 0 | 0.0000 |
| GBR | 182 | 0 | 0.0000 |
| GIH | 206 | 0 | 0.0000 |
| GWD | 226 | 0 | 0.0000 |
| IBS | 214 | 0 | 0.0000 |
| ITU | 203 | 1 | 0.0049 |
| JPT | 208 | 0 | 0.0000 |
| KHV | 192 | 6 | 0.0303 |
| LWK | 198 | 0 | 0.0000 |
| MSL | 170 | 0 | 0.0000 |
| MXL | 128 | 0 | 0.0000 |
| PEL | 170 | 0 | 0.0000 |
| PJL | 192 | 0 | 0.0000 |
| PUR | 208 | 0 | 0.0000 |
| STU | 204 | 0 | 0.0000 |
| TSI | 214 | 0 | 0.0000 |
| YRI | 216 | 0 | 0.0000 |

*ACB: African Carribbeans in Barbados. ASW: Americans of African Ancestry in SW USA. BEB: Bengali from Bangladesh. CDX: Chinese Dai in Xishuangbanna. CEU: Utah Residents (CEPH) with north and westren European Ancestry. CHB: Han Chinese in Bejing, China. CHS: Southern Han Chinese. CLM: Colombians from Medellin, Colombia. ESN: Esan in Nigeria. FIN: Finnish in Finland. GBR: British in England and Scotland. GIH: Gujarati Indian from Houston, Texas. GWD: Gambian in Western Division in the Gambia. IBS: Iberian Population in Spain. ITU: Indian Telugu from the UK. JPT: Japanese in Tokyo, Japan. KHV: Kinh in Ho Chi Minh City, Vietnam. LWK: Luhya in Webuye, Kenya. MSL: Mende in Sierra Leone. MXL: Mexican Ancestry from Los Angeles, USA. PEL: Peruvians from Lima, Peru. PJL: Punjabi from Lahore, Pakistan. PUR: Puerto Ricans from Puerto Rico. STU: Sri Lankan Tamil from the UK. TSI: Toscani in Italia. YRI: Yoruba in Ibadan, Nigeria.
